# The relation between mental health problems and future violence among detained male juveniles

**DOI:** 10.1186/s13034-019-0264-5

**Published:** 2019-01-12

**Authors:** Olivier F. Colins, Thomas Grisso

**Affiliations:** 1 Department of Child and Adolescent Psychiatry, Curium-Leiden University Medical Center, Endegeesterstraatweg 27, AK 2342 Leiden, The Netherlands; 2 Center for Criminological and Psychosocial Research, Örebro University, Örebro, Sweden; 3 Affiliated Researcher Academic Workplace Forensic Care for Youth (Academische Werkplaats Forensische Zorg Voor Jeugd), Zutphen, The Netherlands; 4 Department of Special Needs Education, Ghent University, Ghent, Belgium; 5 Department of Psychiatry, University of Massachusetts Medical School, Worcester, MA USA

**Keywords:** Mental health, Antisocial, Detained, Violence recidivism, Risk assessment

## Abstract

**Background:**

Detention personnel may assume that mental health problems heighten the likelihood of future violence in detained youth. This study explored whether brief mental health screening tools are of value for alerting staff to a detained youth’s potential for future violent offending.

**Method:**

Boys (n = 1259; Mean age = 16.65) completed the Massachusetts Youth Screening Instrument-Second Version (MAYSI-2) and the Strengths and Difficulties Questionnaire (SDQ) as part of a clinical protocol. Official records were collected to index past and future violent offending.

**Results:**

A few significant positive and negative relationships between MAYSI-2 and SDQ scale scores and future violent offending were revealed, after controlling for age, past violent offending, and follow-up time. These relations were almost entirely dissimilar across the ethnic groups, even to the extent of finding opposite relations for boys in different ethnic groups.

**Conclusions:**

The small number of relations and their small effect sizes suggest little likelihood that screening for mental health problems in boys who are detained in the Netherlands offers any potential for identifying youth at risk for committing future violent crimes. The current findings also suggest that ethnic differences in the relation between mental health problems and future criminality must be considered in future studies.

**Electronic supplementary material:**

The online version of this article (10.1186/s13034-019-0264-5) contains supplementary material, which is available to authorized users.

## Background

Based on national studies in several countries, youth retained in juvenile justice facilities display high levels of mental health problems, often so severe that they meet criteria for at least one psychiatric disorder (for a review see: [1, 2]). In the U.S. [3, 4] and the Netherlands [5], standardized mental health screening procedures have become routine upon entry into juvenile justice programs to determine the need for emergency mental health services and for additional comprehensive assessment. The present study explored whether brief mental health screening tools, when used shortly after a youth’s entry into detention settings, might be of value for alerting staff to a youth’s potential for future violent offending, thus suggesting the need for more definitive evaluation for risk of harm.

Mental health screening tools, of course, are not developed for that purpose. They are designed to identify youth whose mental health symptoms suggest the need for further assessment to determine need for mental health services [6]. But if these tools generate mental health screening scores that are related to future violent behavior, this could be of value. Routine evaluation for risk of aggression is not standard practice immediately up a youth’s entry to detention centers, which might be unfortunate since the juvenile justice system has not only an obligation to meet the mental health needs of youth in its custody, but also to protect other youth, detention staff, and the community from harm.

Theory and research on the general relation of mental disorders and violent offending among youth offer mixed expectations regarding a mental health screening instrument’s potential capacity to predict one from the other. Some of the common risk factors for youth offending (e.g., irritability, impulsiveness, substance use) are also symptoms of disorders of youth (e.g., related to depression, traumatic stress, attention deficit disorder, or substance use disorders). Consistent with this, some studies have found that symptoms of psychiatric disorders co-vary with reliable risk predictors of violence (e.g., [7, 8]). Other studies have found a small to moderate positive association between psychiatric disorder and future violent offending, although with much inconsistency in the specific disorder or disorder categories that were related to future violence (e.g., [9–11]). Therefore, one might expect to find at least modest relations with future violence because some scales of mental health screening tools include items referring to alcohol and drug use, impulsivity or irritability or anger. This would not suggest that mental health screening tools can serve as strong predictors of violence for judicial decision-making purposes. If modest relations were found, the value would be in the tools’ ability to *alert* detention staff to engage in further in-depth violence risk assessment to determine whether the youth offers a prospect of danger to staff, other youth in detention or, if released, to others in the community.

However, only a few studies have examined this relationship among criminal justice-involved youth using screening tools. For example, using the Massachusetts Youth Screening Instrument-Second Version (MAYSI-2; [12]), anger and thought disturbance were related to later aggression during detention [13, 14], whereas alcohol/drug use and anger were not predictive of violence after being released [15, 16]. Using the Strengths and Difficulties Questionnaire (SDQ; [17]), others found that mental health symptoms (e.g. emotion problems and hyperactivity) were not related to violent recidivism [18]. Unfortunately, firm conclusions are precluded because the studies differed greatly in the variety of mental health symptoms that were considered (e.g. the aforementioned MAYSI-2 studies merely used one or two out of the six clinical scales), the outcome of interest (violence during or after detention), and the control variables included in the analyses. To better inform the science and practice of forensic mental health assessments [19], the present study examined the relation of mental health screening data that were gathered in the context of a clinical protocol for all youth entering two all-male youth detention centers in the Netherlands. The data base included substantial numbers of detained youth from three ethnic origins (Dutch, Moroccan and Surinamese/Antillean). We examined the relation of mental health symptoms and future violence in these three groups (and in a fourth group of “other” ethnicity) separately, for four reasons. First, past studies indicated differences between various ethnic groups in levels of mental health problems (e.g., [5, 20]) and recidivism (e.g., [21]). Second, people of different ethnic origins may respond to mental health screening and assessment tools differently because of variations in openness to acknowledging symptoms (e.g., [22]). Third, prior work suggested that the relation between mental health problems and future criminality differ across ethnic groups [18, 23]. Fourth, notwithstanding that two of the ethnic groups (i.e., Dutch and Surinamese/Antillean) were quite specific to juvenile justice settings in the country in which the data were obtained, examination of ethnic differences was expected to contribute more generally to the literature on the relevance of ethnicity in mental health screening and violent risk assessment [24].

Specifically, the present study used two mental health screening tools (one supplementing the other) to explore whether their scores were related to future arrests for violent behavior. We hypothesized that some symptoms identified on the screening tools would be related modestly to future arrests for violent behavior, but that those relations would vary (in type of symptoms and strength of the relations) for different ethnic groups. Our efforts were exploratory in the sense that we did not form hypotheses regarding specific symptoms or specific ethnic differences.

## Methods

### Participants

Participants were adolescent and young adult males, aged 12 to 25 years (M = 16.65; SD = 1.43). Most were 15–17 years (80.5%), while the remainder being 12–14 (8.5%) and 18–25 (11.0%). They were in custody in two large youth detention centers (YDCs) in urban areas in the Netherlands, where the MAYSI-2 and SDQ were given as a routine part of the detention centers’ intake processes, to all entering youth consecutively between May 2008 and December 2012 (for details, see for example: [25, 26]). For the current study, data were used from 1259 detained male adolescents who completed the mental health screening and assessment protocols and for whom official criminal records were available. Regarding ethnicity (as defined below), 22.6% of the boys were of Dutch origin, 25.5% of Moroccan origin, 21.1% of Surinamese/Antillean origin, and 30.0% included a wide variety of ethnic or national origins. These percentages are consistent with those presented in prior work with detained boys in the Netherlands (e.g., [18]). For 10 boys (0.8%), information to determine ethnicity was lacking, and these boys were excluded from the study, resulting in total sample of 1249 boys.^1^

### Measures

#### Massachusetts Youth Screening Instrument-Second Version (MAYSI-2 [12])

The MAYSI-2 is a 52-item screening tool in which youth answer questions (yes/no) that sample the presence or absence of symptoms or behaviors related to several areas of emotional, behavioral, and psychological disturbances. The MAYSI-2 was specifically designed and normed for use among youth entering a juvenile justice setting, and can be administered in about 15 min by computer or paper and pencil self-report. Factor analyses indicated that the items produce scores on six clinical scales: Alcohol-Drug Use (8 items), Angry-Irritable (9 items), Depressed-Anxious (9 items), Somatic Complaints (6 items), Suicide Ideation (5 items), and Thought Disturbance (for boys only; 5 items); and one non-clinical scale (Traumatic Experiences; 5 items). There is no MAYSI-2 total score as the test was not intended to measure a broader construct such as mental distress or emotional disturbance [12]. None of the scales were intended to be diagnostic of DSM-5 mental disorders, merely to identify symptoms suggesting the need for further assessment (e.g. [27]). Each clinical MAYSI-2 scale has a “Caution” cutoff empirically developed to identify youth who might be in need of clinical attention [28]. Each clinical scale also has a “Warning” cutoff identifying scores obtained by the top 10% of youth in the original Massachusetts normative sample [12], flagging youth who are most in need of clinical attention.

The present study used the official Dutch version of the MAYSI-2 [29] which was developed using translation and back-translation procedures. The Dutch MAYSI-2 has been shown to have good psychometric properties in terms of factor structure, internal consistency, and construct validity [5, 25, 27] in youth being detained in the Netherlands, including detained youth from Dutch, Moroccan, Surinamese/Antillean, and Mixed ethnicity.^2^ The MAYSI-2 was introduced in various European countries in the past eight years, including the Netherlands (see: http://www.inforsana.eu). Pending further information being developed in Europe, clinicians are guided to use the cut-off scores developed for use in the U.S. [12, 30]. The current study relied on the six raw clinical MAYSI-2 scale scores and U.S. based Caution cut-offs (unless otherwise stated).

The Cronbach’s alpha (α) and mean inter-item correlation (MIC) for the six clinical MAYSI-2 scales in the total sample (N = 1249) were as follows: Alcohol/Drug Use (α = 0.84; MIC = 0.40); Angry-Irritable (α = 0.76; MIC = 0.27); Depressed-Anxious (α = 0.67; MIC = 0.19); Somatic Complaints (α = 0.58; MIC = 0.19); Thought Disturbance (α = 0.50; MIC = 0.17); and Suicide Ideation (α = 0.77; MIC = 0.41). Of note, α can be interpreted as follows: < 0.60 = insufficient; 0.60–0.69 = marginal; 0.70–0.79 = acceptable; 0.80–0.89 = good, and 0.90 or higher = excellent [31]. Because α penalizes shorter scales, [32] we also presented MIC values, which is considered to be a more straightforward indicator of the internal consistency of a scale than α, and should be at minimum in the range of 0.15 to 0.50 to be considered adequate [33]. Additional file 1: Part 1, presents α and MIC values for the six MAYSI-2 scales across the four ethnic groups.

#### The Strength and Difficulties Questionnaire self-report version (SDQ [17])

The SDQ is a self-report and third-party informant (parent and teacher) screening instrument for psychosocial functioning of children and adolescents. The current study used the self-report version. The SDQ has five subscales, each with five items offering three response categories (*Not true* = 0, *Somewhat true* = 1*, Certainly true* = 2), has been used with detained youth in prior research (e.g., [18, 26]), and is used internationally (e.g., [34–36]). The present study used two SDQ scales—Conduct Problems, and Hyperactivity—that are not covered by the MAYSI-2. “Borderline Cut-off” scores for these two scales are 4 and 6, respectively [37]. The current study used the raw scores and borderline cut-offs unless otherwise specified. The α and MIC for the two SDQ scales in the total sample (N = 1249) were as follows: Conduct Problems (α = 0.55; MIC = 0.22) and Hyperactivity (α = 0.79; MIC = 0.43). Of note, prior work revealed that αs for these latter two scales ranged from 0.47 to 0.60 (Conduct Problems), and from 0.66 to 0.67 (Hyperactivity) in epidemiological sample of British adolescents [38] and a community sample of Dutch adolescents [39]. Additional file 1: Part 1, presents α and MIC values for these two SDQ scales across the four ethnic groups.

#### Omnibus variable

Using the MAYSI-2 and SDQ, we also created an “omnibus variable” that reflects the number of times participants were at or above the Caution (MAYSI-2) or Borderline Cut-off (SDQ) on the eight scales being used to measure eight different types of mental health problems (i.e., six MAYSI-2 and two SDQ scales). This omnibus variable, from here onwards referred to as “Omnibus Mental Health Problems” (theoretical range 0–8), was intended to be indicative of the severity or multiplicity of mental health problems. The percentages of boys at or above various cut-off scores can be retrieved from Additional file 2: Part 2.

#### Violent criminality

Violent arrest was defined as any offense involving physical harm to another person (e.g., manslaughter, theft with violence, and sex offenses). Data were gathered based on the General Documentation Registry (GDR) of the Ministry of Justice Court Documentation Service of the Netherlands. The Registry contains information on the number, time, and nature of all criminal cases registered at the Public Prosecutor’s Office, including their adjudication. We used all registered cases, regardless of their adjudication. Specifically, in addition to cases that ended in a guilty ruling, cases that ended in a prosecutorial waiver or an acquittal were also included when reconstructing the respondents’ criminal career. Data include all such information from age 12, which is the minimum age of legal responsibility in the Netherlands, to the respondents’ age on June 30th 2013, which represents the end of the follow-up period for this study. The variable Past Violent Arrests refers to the number of violent arrests before the completion of screening (i.e. shortly after detention intake, see Procedure). The variable Future Violent Arrests refers to the number of violent arrests in the follow-up period, that is the weeks between completion of screening and June 30th 2013.^3^ The percentage of youth with at least one prior violent arrest was 76.1% for Dutch boys, 74.1% for Moroccan boys, 86.1% for Surinamese/Antillean boys, and 79.1% of Mixed Origin boys. For future violent arrest these percentages were 27.5% (Dutch), 34.9% (Moroccan), 41.4% (Surinamese/Antillean), and 32.8% (Mixed Origin).

#### Ethnic background

Based on the Dutch standard classification of ethnic groups [40] and in line with prior work from the Netherlands (e.g., [5]), a participant was categorized as “Moroccan” or “Surinamese/Antillean” when the adolescent himself and/or at least one parent had been born in Morocco or Surinam/Dutch Antilles, respectively. When both parents were of different non-Dutch origin, we used the mother’s country of birth to determine the child’s ethnicity. Participants were classified as Dutch when both parents and the child were born in the Netherlands. All other participants were assigned to the “Mixed Origin” group, implying not “mixed identity” for any one participant, but simply a group comprised of mixed ethnic origins.

### Procedure

The MAYSI-2 and SDQ were administered on a standalone computer within a few days after detention entry (Mean number of days = 3.3, SD = 5.6) in the presence of non-clinical personnel, to all youth entering YDCs. Assistance was available at request (e.g., if the youth did not understand a question). When reading abilities were insufficient, the questionnaires were read to the youth. Youth were made aware that the mental health screening and assessment were part of the YDCs’ clinical protocol and that all the outcomes from this protocol were available to YDCs personnel (e.g., clinicians) and could be included in their file. Through standardized oral and written information provided by the YDCs upon start of detention, youth and their parents/care-takers were informed that the mental health screening and assessment outcomes would be used for scientific research, unless they declined (passive informed consent). They were also informed that, if they did not decline, their information would be transferred anonymously to the researchers, so that information could not be traced back to them. The Medical Ethical Review Board of the Leiden University Medical Center deemed study protocols to be exempt from review because data were collected by the YDCs as part of a clinical protocol and for clinical purposes.

### Data-analyses

Multivariate Poisson regression analyses (with 95% confidence intervals [CI]) were conducted to examine the relation between mental health problems and future violent arrests. These analyses were performed in two ways. First, we examined the relation of each MAYSI-2 and SDQ scale score to violent arrests (called the “bivariate model”). Second, we examined each scale’s relation to violent arrests when all other scales were added to the analysis, together with three control variables, being: age (at detention entry), number of past violent offenses, and follow-up time (called the “multivariate model”). These control variables are important to consider because age is inversely related to criminal recidivism (e.g., [41]), because past violent offending is a robust predictor of future violence (e.g., [42]), and because some research has suggested that mental health problems may lose their value for predicting future violent offending after controlling for prior violent offending (e.g., [7]). It is also important to account for differences in the time participants had to commit new violent crimes. Therefore, follow-up time was used as a control variable as well. To avoid finding significant differences due simply to random error when computing large numbers of tests, we discounted any significant relations as “uninterpretable” (nullified) if 20% or fewer significant relations were revealed within an ethnic group. Specifically, this implies that when running nine tests in one ethnic group (i.e., eight single scale models plus one control model) at least 2 or more significant effects must be revealed. This is a conservative criterion, as “chance” findings of significance by random error in multiple comparisons usually are interpreted as 1 in 20 (5% of comparisons) (e.g., [43]).

Next, the aforementioned analyses were repeated using the Omnibus Mental Health Problems variable instead of the raw MAYSI-2 and SDQ scores. This omnibus variable (i.e. number of times at or above MAYSI-2 and SDQ cut-offs) may be appealing for clinicians who want to identify youth with comorbid mental health problems for decision making related to screening, and may prefer to use dichotomies rather than dimensional scores [44]. However, these cut-off scores derived in the U.S. (MAYSI-2) or Britain (SDQ) might not be optimal to identify detained youth in the Netherlands with elevated mental health problems.

To circumvent the potential problem that our Omnibus variable is based on a less-than-optimal cut-off score, we also performed latent profile analyses (LPA) using Mplus 6.1 [45] to identify distinct subgroups based on their permutations of raw MAYSI-2 and SDQ scale scores. LPA is a data-driven, person-oriented, model-based clustering technique to assign youth to mutually exclusive subgroups and uses statistical criteria to compare models to identify the optimal number of groups to retain [46]. Technical details for LPA are provided in Additional file 3: Part 3. In this study, the six raw MAYSI-2 and two raw SDQ scale scores were used as the clustering variables in LPA. The outcome of these LPA will be used for comparison and predictive purposes. All analyses were performed separately for each ethnic group. SPSS 23.0 was used, unless otherwise specified, with *p* < 0.05 as an indicator of statistical significance.

## Results

### Descriptive information

Mean scores and standard deviations are presented in Table 1. Moroccan boys scored lower than Dutch boys on all eight scales and also lower than Surinamese/Antillean and Mixed Origin boys on most of these scales.^4^ Post hoc tests also showed that Dutch and Moroccan boys were not significantly different in the number of future violent arrest, though Dutch boys had significantly fewer future violent arrests than Surinamese/Antillean boys.

**Table 1:** Distribution of mental health problems, future violent arrest, and control variables across ethnic groups

	Dutch (n = 284)		Moroccan (n = 321)		Surin/Ant (n = 266)		Mixed Origin (n = 378)	
	M	(SD)	M	(SD)	M	(SD)	M	(SD)
Alcohol/drug use	2.50^a^	(2.44)	0.52^b^	(1.30)	1.39^c^	(1.97)	1.29^c^	(1.97)
Angry-irritable	2.90^a^	(2.43)	1.34^b^	(1.89)	2.26^c^	(2.11)	2.03^c^	(2.12)
Depressed/anxious	1.50^a^	(1.58)	0.85^b^	(1.37)	1.35^ac^	(1.55)	1.33^ac^	(1.70)
Somatic complaints	1.99^a^	(1.45)	1.45^b^	(1.45)	1.72^ab^	(1.40)	1.78^a^	(1.43)
Thought disturbances	0.48^a^	(0.80)	0.27^b^	(0.67)	0.40^ab^	(0.76)	0.33^ab^	(0.73)
Suicide ideation	0.48^a^	(1.07)	0.09^b^	(0.43)	0.26^c^	(0.82)	0.28^c^	(0.83)
Conduct problems	2.53^a^	(1.88)	1.69^b^	(1.56)	2.13^c^	(1.67)	2.03^c^	(1.70)
Hyperactivity	4.78^a^	(2.41)	2.14^b^	(2.28)	2.90^c^	(2.27)	3.09^c^	(2.36)
Omnibus variable	2.32^a^	(2.02)	0.92^b^	(1.48)	1.44^c^	(1.66)	1.40^c^	(1.72)
Number of future violent arrests	0.40^a^	(0.76)	0.57^ab^	(0.96)	0.63^b^	(0.94)	0.49^ab^	(0.86)
Age	16.83^a^	(1.46)	16.70^ab^	(1.32)	16.62^ab^	(1.51)	16.50^b^	(1.44)
Number of past violent arrests	1.21^a^	(1.04)	1.32^ab^	(1.26)	1.54^b^	(1.18)	1.34^ab^	(1.10)
Follow-up time (weeks)	149.4^a^	(74.34)	134.2^ab^	(69.37)	145.1^ab^	(71.61)	130.7^b^	(72.38)

Surin/Ant, Surinamese/Antillean. Means with different superscripts refer to significant group differences, based on Games-Howell correction for all but two variables: age and follow-up time. For these two latter variables Bonferroni correction was used because the homogeneity of variance criterion was met; the difference between Dutch and Mixed Origin boys in Thought Disturbances (*p* = 0.054), and between Dutch and Moroccan boys in number of future violent arrests (*p* = 0.06) almost reached statistical significance. Differences in Follow-Up Time almost reached significance when comparing Dutch with Moroccan (*p* = 0.06) and Surinamese/Antillean with Mixed Origin boys (*p* = 0.07)

### Variable-oriented analyses: mental health problems and future violent arrests

As shown in Table 2 significant effects were found on two (Dutch), one (Moroccan), four (Surinamese/Antillean), and seven (Mixed Origin) out of nine tests, rendering these effects “interpretable” according to our random error criterion in all but one ethnic group (Moroccan boys). Among Dutch boys, Depressed-Anxious was positively related to future violent arrests in the multivariate model. Yet, among Surinamese/Antillean boys, Depressed-Anxious was negatively related to future violent arrests (bi- and multivariate models), whereas Somatic Complaints and Suicide Ideation were also negatively related to future violent arrests among these boys, though only in the bivariate models. Among Mixed Origin boys, positive relations with future violent arrests were revealed for Angry-Irritable and Alcohol/Drug use (bi- and multivariate models), and for Depressed/Anxious, Suicide Ideation, and Conduct Problems (bivariate models).

**Table 2:** Mental health screening scores as predictors of total number of future violent arrests

Sample	Scale	Bivariate model	Multivariate model^a^
		EXP(B); 95% CI	EXP(B); 95% CI
Dutch	Alcohol/drug use	1.04 (0.96; 1.12)	1.06 (0.98; 1.16)
	Angry-irritable	1.01 (0.94; 1.09)	0.98 (0.87; 1.10)
	Depressed-anxious	1.07 (0.96; 1.19)	*1.19* (1.01; 1.42)
	Somatic complaints	1.01 (0.89; 1.14)	1.00 (0.86; 1.16)
	Thought disturbances	0.88 (0.69; 1.14)	*0.71* (0.52; 0.95)
	Suicide ideation	1.02 (0.86; 1.21)	1.02 (0.83; 1.24)
	Conduct problems	0.97 (0.88; 1.07)	0.98 (0.85; 1.11)
	Hyperactivity	0.94 (0.86; 1.01)	0.91 (0.83; 1.01)
Moroccan	Alcohol/drug use	1.06 (0.96; 1.18)	0.98 (0.86; 1.11)
	Angry-irritable	1.03 (0.95; 1.10)	1.01 (0.89; 1.14)
	Depressed-anxious	1.01 (0.91; 1.12)	1.08 (0.91; 1.28)
	Somatic complaints	*1.12* (1.02; 1.23)	1.11 (0.99; 1.23)
	Thought disturbances	0.96 (0.77; 1.21)	0.96 (0.74; 1.29)
	Suicide ideation	0.76 (0.48; 1.22)	0.57 (0.31; 1.05)
	Conduct problems	1.07 (0.98; 1.17)	1.03 (0.91; 1.17)
	Hyperactivity	0.99 (0.93; 1.06)	0.99 (0.91; 1.08)
Surinamese/Antillean	Alcohol/drug use	0.95 (0.88; 1.03)	0.96 (0.87; 1.05)
	Angry-irritable	0.97 (0.90; 1.04)	1.08 (0.97; 1.20)
	Depressed-anxious	*0.82* (0.72; 0.92)	*0.80 (*0.68; 0.95)
	Somatic complaints	*0.87* (0.77; 0.98)	0.97 (0.85; 1.10)
	Thought disturbances	0.93 (0.75; 1.15)	1.04 (0.80; 1.31)
	Suicide ideation	*0.66* (0.47; 0.93)	0.82 (0.58; 1.14)
	Conduct problems	1.00 (0.91; 1.09)	0.94 (0.84; 1.06)
	Hyperactivity	1.02 (0.96; 1.09)	1.06 (0.99; 1.14)
Mixed Origin	Alcohol/drug use	*1.12* (1.05; 1.19)	*1.10* (1.01; 1.19)
	Angry-irritable	*1.17* (1.11; 1.25)	*1.14* (1.03; 1.26)
	Depressed-anxious	*1.11* (1.03; 1.20)	0.94 (0.84; 1.06)
	Somatic complaints	1.06 (0.96; 1.17)	0.97 (0.86; 1.09)
	Thought disturbances	1.10 (0.92; 1.31)	1.00 (0.81; 1.23)
	Suicide ideation	*1.23* (1.08; 1.40)	1.07 (0.91; 1.26)
	Conduct problems	*1.14* (1.05; 1.22)	0.95 (0.85; 1.06)
	Hyperactivity	1.01 (0.95; 1.07)	0.94 (0.88; 1.02)

The bivariate model includes only one scale; the multivariable model simultaneously includes all eight scales and age; overall, deviance tests provided values close to 1.00, thereby suggesting that there were no problems with under- or overdispersion (range of values for the three models: Dutch: 1.06–1.13; Moroccan: 1.01–1.37; Surinamese/Antillean: 1.09–1.29; Mixed Origin: 1.02–1.22); italicised values are significant at p < .05

^a^Of the three control variables included in the multivariate model, the following were significantly related to the total number of future violent arrests among Dutch boys: Follow-Up Time [Exp(B): 1.01; CI 1.002–1.01] and number of past violent arrests [Exp(B): 1.20; CI 1.002–1.44]; among Moroccan boys: Follow-Up Time [Exp(B): 1.01; CI 1.002–1.01] and number of past violent arrests [Exp(B): 1.01; CI 1.008–1.012]; among Surinamese/Antillean boys: Follow-up Time [Exp(B): 1.007; CI 1.005–1.010]; and among Mixed Origin boys: Age [Exp(B): 1.01; CI 1.002–1.01] and Follow-up Time [Exp(B): 1.006; CI 1.004–1.010]

Though not shown in Table 2, significant effects for the Omnibus Mental Health Problems variable were revealed in two ethnic groups. Specifically, this variable was negatively related to future violent arrests among Surinamese/Antillean boys (multivariate model: Exp(B): 0.89; CI 0.80; 0.99) but positively among Mixed Origin boys, (bivariate model: Exp(B): 1.14; CI 1.06; 1.23). Details are available upon request.

### Person-oriented analyses: mutually exclusive subgroups and future violent arrests

#### Subgroup identification

**Fig. 1:**
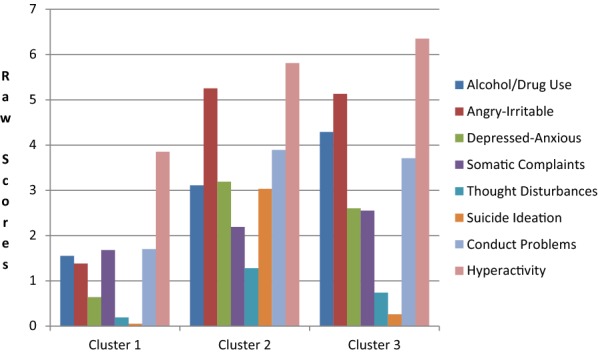
Mean MAYSI-2 and SDQ scale scores for three clusters of Dutch boys

Statistics presented in Additional file 4: Part 4 shows that a 3-subgroup model best fit the data for Dutch boys. As shown in Table 3 and Fig. 1, Cluster 1 (59.9% of the Dutch boys) was characterized primarily by relatively lower MAYSI-2 and SDQ scores. Clusters 2 (12.7% of Dutch boys) and 3 (27.5% of Dutch boys) were significantly higher on all MAYSI-2 and SDQ scales than Cluster 1, and differed from each other in two ways: Cluster 2 had a lower Alcohol/Drug Use score, but higher Thought Disturbance and Suicide Ideation scores than Cluster 3. For the other three ethnic groups, a 2-subgroup model best fit the data. Table 4 shows that 15.3% of Moroccan, 5.6% of Surinamese/Antillean and 19.0% of Mixed Origin boys were assigned to a cluster that had significant higher scores on all eight scales than the boys who were assigned to the other cluster. These 2-cluster solutions indicate that the only data-driven distinction that could be made within these three ethnic groups was between subgroups with higher (Cluster 2) and lower (Cluster 1) levels of mental health problems.

**Table 3:** Distribution of mental health problems as clustering variables, the omnibus mental health problems variable, and future violent arrests, and control variables across three clusters of Dutch boys

	Cluster 1 (n = 170)	Cluster 2 (n = 36)	Cluster 3 (n = 78)	Pair-wise comparisons
	M (SD)	M (SD)	M (SD)	M (SD)
Alcohol/drug use	1.55 (1.96)	3.11 (2.33)	4.29 (2.36)	1 < 2, 3; 2 < 3
Angry-irritable*	1.38 (1.36)	5.25 (2.05)	5.13 (1.73)	1 < 2, 3
Depressed-anxious*	0.64 (0.82)	3.19 (1.53)	2.60 (1.56)	1 < 2, 3
Somatic complaints*	1.68 (1.29)	2.19 (1.43)	2.55 (1.60)	1 < 3
Thought disturbances*	0.19 (0.45)	1.28 (0.97)	0.74 (0.95)	1 < 2, 3; 3 < 2
Suicide ideation*	0.05 (0.21)	3.03 (1.00)	0.26 (0.44)	1 < 2, 3; 3 < 2
Conduct problems*	1.70 (1.38)	3.89 (1.88)	3.71 (1.89)	1 < 2, 3
Hyperactivity	3.85 (2.08)	5.81 (2.21)	6.35 (2.18)	1 < 2, 3
Omnibus variable*	0.99 (0.92)	5.0 (1.40)	4.0 (1.5)	1 < 2, 3; 3 < 2
Future violent arrests	0.39 (0.79)	0.44 (0.81)	0.38 (0.71)	
Age	16.85 (1.46)	16.73 (2.03)	16.84 (1.12)	
Past violent arrests	1.12 (0.97)	1.11 (1.39)	1.45 (0.97)	
Follow-up time (weeks)	148.5 (73.20)	167.44 (76.8)	143.14 (75.31)	

Pair-wise comparisons based on Bonferroni unless otherwise specified

* Pair-wise comparisons based on Games–Howell

**Table 4:** Distribution of mental health problems as clustering variables, the omnibus mental health problems variable, future violent arrests, and control variables within moroccan, surinamese/antillean, and mixed origin boys

Clustering variables	Moroccan			Surinamese/Antillean			Mixed Origin		
	Cluster 1 (n = 272)	Cluster 2 (n = 49)		Cluster 1 (n = 251)	Cluster 2 (n = 15)		Cluster 1 (n = 279)	Cluster 2 (n = 99)	
Alcohol/drug use	0.32 (0.94)	1.63 (2.19)	1 < 2	1.31 (1.91)	2.80 (2.45)	1 < 2	0.63 (1.26)	3.13 (2.39)	1 < 2
Angry-irritable	0.71 (1.02)	4.88 (1.69)	1 < 2	2.07 (1.95)	5.47 (2.30)	1 < 2	1.07 (1.22)	4.73 (1.77)	1 < 2
Depressed-anxious	0.49 (0.86)	2.84 (1.90)	1 < 2	1.18 (1.31)	4.33 (2.29)	1 < 2	0.69 (1.09)	3.14 (1.80)	1 < 2
Somatic complaints	1.15 (1.21)	3.06 (1.60)	1 < 2	1.65 (1.34)	2.93 (1.83)	1 < 2	1.47 (1.22)	2.68 (1.62)	1 < 2
Thought disturbances	0.11 (0.34)	1.18 (1.17)	1 < 2	0.33 (0.66)	1.53 (1.25)	1 < 2	0.15 (0.42)	0.83 (1.09)	1 < 2
Suicide ideation	0.01 (0.12)	0.51 (0.96)	1 < 2	0.09 (0.31)	3.13 (1.25)	1 < 2	0.08 (0.35)	0.87 (1.35)	1 < 2
Conduct problems	1.30 (1.15)	3.84 (1.80)	1 < 2	2.08 (1.67)	3.00 91.47)	1 < 2	1.41 (1.19)	3.78 (1.71)	1 < 2
Hyperactivity	1.58 (1.73)	5.24 (2.46)	1 < 2	2.79 (2.22)	4.73 (2.40)	1 < 2	2.46 (2.08)	4.87 (2.20)	1 < 2
Omnibus variable	0.40 (0.68)	3.80 (1.43)	1 < 2	1.22 (1.37)	5.00 (2.00)	1 < 2	0.59 (0.82)	3.68 (1.54)	1 < 2
Future violent arrests	0.58 (0.97)	0.51 (0.92)		0.66 (0.96)	0.13 (0.35)	1 > 2	0.39 (0.70)	0.76 (1.17)	1 < 2
Age	16.75 (1.31)	16.40 (1.36)		16.62 (1,53)	16.61 (1.22)		16.52 (1.61)	16.50 (1.44)	
Past violent arrests	1.38 (1.32)	0.96 (0.79)	1 > 2	1.57 (1.20)	1.07 (0.70)		1.29 (1.03)	1.48 (1.27)	
Follow-up time (weeks)	134.1 (69.03)	134.6 (79.96)		145.8 (71.18)	133.4 (80.06)		121.9 (68.67)	155.4 (76.82)	1 < 2

#### Subgroups and future violent arrests

Among Dutch and Moroccan boys, no significant differences in risk for future violent arrests emerged between the three (Dutch boys) or two (Moroccan boys) clusters, neither in the bivariate nor the multivariate model (details available upon request). Surinamese/Antillean boys with higher levels of mental health problems (Cluster 2) had a significantly lower risk for future violent arrests [bivariate model: Exp(B) = 0.20; CI 0.05–0.82] than Surinamese/Antillean boys with lower levels of mental health problems (Cluster 1), a finding that remained after controlling for age, follow-up time and the total number of past violent arrests (multivariable model: Exp(B) = 0.22; CI 0.05–0.89). Mixed Origin boys with higher levels of mental health problems (Cluster 2) had a significantly elevated risk for future violent arrests [bivariate model: Exp(B) = 1.92; CI 1.43–2.58] than Mixed Origin boys with lower levels of mental health problems (Cluster 1), a finding that remained after controlling for age, follow-up time and the total number of past violent arrests (multivariate model: Exp(B) = 1.43; CI 1.06–1.95).

## Discussion

This study explored whether brief mental health screening tools, when used in youth detention settings, might be of value for staff to identify detained boys at risk for future violence, thus suggesting the need for more definitive evaluation for risk of harm. We found a few significant relationships between MAYSI-2/SDQ scales and future violent arrests, and some were consistent with various past theoretical speculations or studies. For example, the negative relation between Thought Disturbances and future violent arrests in Dutch boys is consistent with prior work on the link between psychotic-like symptoms and future violence arrests among criminal justice-involved individuals (e.g., [18]). Also, both the positive (Dutch boys) and negative (Surinamese/Antillean boys) prospective relation between Depressed-Anxious and future violent arrest are consistent with theoretical notions that (i) depression in boys is often expressed by aggressive behaviors, which may lead to increased interpersonal conflicts and subsequently increase the risk of contact with the juvenile justice system [47–49], and (ii) depressive feelings, anxiousness and nervousness may protect against future violence because of apathy, lower energy levels and avoiding situations that cause tension [50, 51].

The most appropriate interpretation of our findings, though, looks to the small number of relations and their small effect sizes. In this light, our results suggest little likelihood that screening for mental health problems in boys who are detained in the Netherlands offers any potential for identifying youth at risk for future violent arrests. Prior work with the SDQ in the Netherlands [18] and the MAYSI-2 in the U.S. (e.g., [15]) also did not reveal any consistent relation with officially registered future violent crimes after release to the community, suggesting that our findings are not sample- and country-specific. Possibly the strongest message is that when significant relations between mental health problems and future violence were found, they were almost entirely dissimilar across the four ethnic groups, even to the extent of finding opposite relations for boys in different ethnic groups. This is consistent with some prior work [18, 23] suggesting that ethnic differences in the relation between mental health problems and future criminality must be considered in future studies.

Strengths of this study include the relatively large number of boys from various ethnic origins who completed well-validated mental health screening tools as part of a clinical protocol, thereby increasing the ecological validity of the findings, and testing the prospective relation between MAYSI-2 and SDQ scores and officially registered future violence using both variable- oriented (Poisson regression) and person-oriented (latent profile analysis) statistical approaches.

Our findings must be interpreted in the context of several limitations. First, both of the tools we used employ youth self-report, and perhaps data from other sources would have found more meaningful relationships. But our purpose was to test the value of data that typically are available at intake to detention centers, and few detention centers have anything other than youths’ self-report during the first few hours or days of their detention. Second, we did not consider institutional misconduct and therefore cannot exclude the possibility that mental health problems, such as thought disturbance and anger-irritability, might predict violence during detention, as was found by others [13, 14, 52]. Screening tools are influenced not only by enduring traits but also by immediate emotional states, and the latter may be more closely related to immediate (in-custody) aggression than to arrests for violence in the distant future (after release). Third, mental health problems were merely assessed shortly after detention entry. It cannot be excluded that the level of mental health problems decreased during detention, for example, because detention staff adequately responded to their mental health problems. Future research, therefore, is warranted to scrutinize if stability and change of mental health problems are related to future violence. Fourth, it must be acknowledged that prior work demonstrated cross-cultural measurement non-invariance of the SDQ self-report version, suggesting that this tool is not suitable for use in cross-cultural comparisons [53]. Since the SDQ has rarely been used in detained adolescents, future factor analytical studies in these youths on the SDQ self-report version are warranted [18]. Fifth, we used official records of past and future arrests for violent offenses, and sometimes youths’ violent behaviors are more extensive than arrest records indicate. This implies that we might have underestimated true violent offending.

The findings in this study have two main implications. First, they suggest that further research explorations of the ability of mental health screening tools to identify youths with future violent tendencies probably will be of little value. Second, we suspect that detention personnel who use mental health screening tools at detention intake already assume that certain scales, such as the MAYSI-2 Angry-Irritable or the SDQ Conduct Problems scales, suggest a heightened likelihood of future aggression. This study discourages detention personnel from making these presumptions, although the results do not rule out the possibility (in light of other past research) of their value for alerting staff to aggressive behavior during the youth’s stay in detention.

## Additional files

**Additional file 1.** Reliability indices for MAYSI-2 and SDQ scales by ethnic group.

**Additional file 2.** Number and percentages of boys at or above various cut-off scores by ethnic group.

**Additional file 3.** Technical details for latent profile analysis.

**Additional file 4.** Model fit statistics from latent profile analyses by ethnic group.

## Abbreviations

YDCs: youth detention centers
MAYSI-2: Massachusetts Youth Screening Instrument-Second Version
SDQ: Strengths and Difficulties Questionnaire
GDR: General Documentation Registry

## Footnotes

1 Earlier work suggested that 5% of the detained youth in the two detention centers declined to take the MAYSI-2 and the SDQ items as part of the clinical protocol [26]. Although we do not know how many boys refused to complete the questionnaires, it can be assumed that almost all of the boys that were detained between May 2008 and December 2012 are represented in the current data base.

2 The MAYSI-2 was designed for use with youth aged 12–17 years. Prior work on the Dutch MAYSI-2 nevertheless showed that support for the internal consistency and validity of the MAYSI-2 scores remained substantially similar when youth older than 18 years of age were included [5].

3 Technically, the registrations are better referred to as ‘criminal justice contacts’ because one could be arrested by the police (e.g., street fight), but quickly be released after interrogation at the police station. In such instance one would be ‘arrested’ but no record in the GDR would appear. The GDR-registrations refer to all criminal cases of which the police have the opinion that they are in need of the public prosecutor’s attention, and thus carry a stronger indication of guilt than do arrests. Yet for reasons of brevity, we will use ‘arrests’ instead ‘of ‘criminal justice contacts’.

4 Because the measurement invariance of the Dutch MAYSI-2 and SDQ across ethnic groups has not yet been tested, results from the between-group comparisons concerning the MAYSI-2 and SDQ should be interpreted with caution and are only provided for exploratory purposes.
