# Supplementary material for: The relation between mental health problems and future violence among detained male juveniles
Source: Child Adolesc Psychiatry Ment Health. 2019 Jan 12;13:4. doi: 10.1186/s13034-019-0264-5 (PMC6330441; doi:10.1186/s13034-019-0264-5)
Supplement: Supplementary file 1 — Additional file 1. Reliability indices for MAYSI-2 and SDQ scales by ethnic group. [file 13034_2019_264_MOESM1_ESM.docx]

**Additional file 1**

| *Table S1*  Reliability Indices for MAYSI-2 and SDQ Scales by Ethnic Group | | | | | | | | |
| --- | --- | --- | --- | --- | --- | --- | --- | --- |
|  | Dutch  (n = 284) | | Moroccan  (n = 321 | | Surin/Ant  (n = 266) | | Mixed Origin  (n = 378) | |
|  | *α* | *MIC* | *α* | *MIC* | *α* | *MIC* | *α* | *MIC* |
| Alcohol/Drug Use | .83 | .37 | .82 | .34 | .82 | .37 | .83 | .38 |
| Angry-Irritable | .77 | .27 | .77 | .28 | .72 | .22 | .74 | .24 |
| Depressed-Anxious | .61 | .15 | .68 | .21 | .63 | .17 | .70 | .21 |
| Somatic Complaints | .54 | .17 | .64 | .24 | .57 | .19 | .55 | .18 |
| Suicide Ideation | .79 | .40 | .64 | .30 | .79 | .45 | .76 | .39 |
| Thought Disturbance | .41 | .11 | .56 | .22 | .47 | .16 | .55 | .21 |
| Conduct Problems | .61 | .24 | .50 | .20 | .46 | .16 | .57 | .23 |
| Hyperactivity | .73 | .34 | .78 | .42 | .73 | .36 | .77 | .40 |
| *Note*. Surin/Ant = Surinamese/Antillean; MIC = mean inter-item correlation | | | | | | | | |
