# Supplementary material for: The relation between mental health problems and future violence among detained male juveniles
Source: Child Adolesc Psychiatry Ment Health. 2019 Jan 12;13:4. doi: 10.1186/s13034-019-0264-5 (PMC6330441; doi:10.1186/s13034-019-0264-5)
Supplement: Supplementary file 2 — Additional file 2. Number and percentages of boys at or above various cut-off scores by ethnic group. [file 13034_2019_264_MOESM2_ESM.docx]

**Additional file 2**

| *Table S2*  *Number and percentages of boys at or above various cut-offs by ethnic group* | | | | |
| --- | --- | --- | --- | --- |
|  | (1)  Dutch  (n = 284) | (2)  Moroccan  (n = 321 ) | (3)  Sur/Ant  (n = 266) | (4)  Mixed  (n = 378) |
| **MAYSI-2** | **n (%)** | **n (%)** | **n (%)** | **n (%)** |
| Above 1 or more | 200 (70.4) | 117 (36.4) | 151 (56.8) | 196 (51.9) |
| Above 2 or more | 131 (46.1) | 56 (17.4) | 76 (28.6) | 103 (27.2) |
| Above 3 or more | 79 (27.8) | 29 (9.0) | 40 (15.0) | 63 (16.7) |
| Above 4 or more | 44 (15.5) | 14 (4.4) | 20 (7.5) | 24 (6.3) |
| Above 5 or more | 19 (6.7) | 4 (1.2) | 6 (2.3) | 14 (3.7) |
| Omnibus MAYSI-2 [Mean(SD)] | 1.68 (1.59) | 0.69 (1.15) | 1.12 (1.35) ^a^ | 1.07 (1.38) ^a^ |
| **SDQ** | **n (%)** | **n (%)** | **n (%)** | **n (%)** |
| Above 1 | 132 (46.5) | 60 (18.7) | 72 (27.1) | 95 (25.4) |
| Above 2 | 49 (17.3) | 13 (4.0) | 13 (4.9) | 29 (7.7) |
| **MAYSI-2 and/or SDQ** | **n (%)** | **n (%)** | **n (%)** | **n (%)** |
| Above 1 or more | 222 (78.2) | 132 (41.1) | 166 (62.4) | 217 (57.4) |
| Above 2 or more | 161 (56.7) | 71 (22.0) | 97 (36.5) | 128 (33.6) |
| Above 3 or more | 113 (39.8) | 51 (12.7) | 56 (21.1) | 82 (21.7) |
| Above 4 or more | 78 (27.5) | 28 (8.7) | 35 (13.2) | 55 (14.5) |
| Above 5 or more | 56 (16.2) | 12 (3.7) | 15 (5.7) | 28 (7.4) |
| Above 6 or more | 26 (9.2) | 8 (2.5) | 7 (2.7) | 12 (3.2) |
| Above 7 or more | 11 (3.9) | 2 (0.6) | 4 (1.6) | 6 (0.3) |

Sur/Ant = Surinamese/Antillean
