# Supplementary material for: The relation between mental health problems and future violence among detained male juveniles
Source: Child Adolesc Psychiatry Ment Health. 2019 Jan 12;13:4. doi: 10.1186/s13034-019-0264-5 (PMC6330441; doi:10.1186/s13034-019-0264-5)
Supplement: Supplementary file 3 — Additional file 3. Technical details for latent profile analysis. [file 13034_2019_264_MOESM3_ESM.docx]

**Additional file 3**

LPA is a model-based cluster analysis technique and is considered as a specific case of finite-mixture modeling (McLachlan & Peel, 2000; Vermunt & Magidson, 2002). Subgroups are identified through maximum likelihood estimation and all the observed indicator variables are continuous (L. K. Muthén & Muthén, 2011). Models that specify different numbers of classes are tested. The Bayesian information criterion (BIC), Akaike information criterion (AIC), Lo-Mendel-Rubin (LMR) statistics, and entropy value are used as statistical criteria to compare models in order to identify the optimal number of groups to retain (Nylund, Asparouhov, & Muthen, 2007). The model with the lowest BIC and AIC values is preferred. The LMR statistic, which is considered to be a likelihood ratio test between models with a different number of latent classes specified, tests *k*–1 classes against *k* classes, and reveals a significant χ^2^ value (*p* < .05) indicating whether the *k*–1 class model is rejected in favor of the *k* class model (Lo, Mendell, & Rubin, 2001). A non-significant χ^2^value (*p* > .05) shows that a model with one fewer class is preferred. Average posterior probabilities of class membership and the entropy value are also taken into consideration to determine the precision of classification and the degree to which the classes are distinguishable, respectively. Average probabilities equal to or greater than .70 imply satisfactory fit (Nagin, 2005), and an entropy value greater than .70 is preferred because it indicates clear classification and greater power to predict class membership (B. Muthén, 2000).

**References**

Lo, Y., Mendell, N. R., & Rubin, D. B. (2001). Testing the number of components in a normal mixture. *Biometrica, 88*, 767-778.

McLachlan, G. J., & Peel, D. (2000). *Finite mixture models*. New York: Wiley, New York.

Muthén, B. (2000). Methodological issues in random coefficient growth modeling using a latent variable framework: Applications to the development of heavy drinking. In J. Rose, L. Chassin, C. Presson, & J. Sherman (Eds.), *Multivariate Applications in Substance use Research* (pp. 113-140). New York: Hillsdale.

Muthén, L. K., & Muthén, B. (2011). Mplus (6.12) [Computer software].

Nagin, D. S. (2005). *Group-based Modeling of Development*. Cambridge: Harvard University Press.

Nylund, K. L., Asparouhov, T., & Muthen, B. (2007). Deciding on the number of classes in latent class analysis and growth mixture modeling. A Monte Carlo simulation study. *Structural equation modeling, 14*, 535-569.

Vermunt, J. K., & Magidson, J. (2002). Latent class cluster analysis. *Applied latent class analysis, 11*, 89-106.
