# Supplementary material for: The relation between mental health problems and future violence among detained male juveniles
Source: Child Adolesc Psychiatry Ment Health. 2019 Jan 12;13:4. doi: 10.1186/s13034-019-0264-5 (PMC6330441; doi:10.1186/s13034-019-0264-5)
Supplement: Supplementary file 4 — Additional file 4. Model fit statistics from latent profile analyses by ethnic group. [file 13034_2019_264_MOESM4_ESM.docx]

**Additional file 4**

| *Table S3*  Model fit statistics from the latent profile analyses by ethnic groups | | | | | | | | | | |
| --- | --- | --- | --- | --- | --- | --- | --- | --- | --- | --- |
| Sample | | # | | n | Probabilities | AIC | BIC | adj BIC | Entr | LMR |
| Dutch | | 2 | | 187/97 | .97/.94 | 8261.10 | 8352.32 | 8273.05 | .86 | <.001 |
|  | | 3 | | 170/36/78 | .97/.98/.92 | 7990.47 | 8114.53 | 8006.72 | .91 | <.01 |
|  | | 4 | | 57/165/41/21 | .93/.96/.96/1.00 | 7825.47 | 7982.38 | 7846.02 | .93 | ns |
| Moroccan | | 2 | | 272/49 | .99/.99 | 14098.00 | 14192.28 | 14112.99 | .98 | <.001 |
|  | | 3 | | 271/7/43 | .99/1.00/.99 | 13629.34 | 13757.57 | 13649.73 | .98 | *ns* |
| Sur/Ant | | 2 | | 251/15 | .99/1.00 | 12372.09 | 12461.68 | 12382.41 | 1.00 | <.05 |
|  | | 3 | | 35/218/13 | .99/1.00/1.00 | 12126.21 | 12248.05 | 12140.25 | .99 | *ns* |
| Mixed Origin | | 2 | | 279/99 | .98/.93 | 10321.89 | 10420.26 | 10340.94 | .90 | <.001 |
|  | | 3 | | 259/95/24 | .97/.93/.98 | 9927.34 | 10061.12 | 9953.25 | .92 | *ns* |
| BIC = The Bayesian information criterion; AIC = Akaike information criterion; LMR = Lo-Mendel-Rubin; Sur/Ant = Surinamese/Antillean | | | | | | | | | | |
|  |  | |  | |  |  |  |  |  |  |
